# Empowering researchers and healthcare professionals in academic publishing: key insights and practical lessons from the British Society of Antimicrobial Chemotherapy (BSAC) workshop 2025

**DOI:** 10.1093/jacamr/dlag082

**Published:** 2026-05-22

**Authors:** Godwin Pius Ohemu, Tsitsi Arikana Mufunde, Tracie Muraya, Emmanuel Essonanjo, Peter Zarb, Rasha Abdelsalam Elshenawy, Neil Powell, Virve I Enne, Riina Rautemaa-Richardson

**Affiliations:** The Global Health Network, Nuffield Department of Medicine, University of Oxford, Old Road Campus, Oxford OX3 7BN, UK; Microbiology Clinical Services, NHS Blood and Transplant, London, UK; Deputy Director for Policy & Strategy, ReAct Africa, Nairobi, Kenya; Department of Biomedical Science, Coventry University, Priority Street, Coventry, West Midlands CV1 5FB, UK; Department of Pathology, University of Malta, Mater Dei Hospital, Msida MSD 2090, Malta; Department of Medicine, School of Health, Medicine and Life Sciences, University of Hertfordshire, Hatfield, UK; Pharmacy Department, Royal Cornwall Hospital, Truro TR1 3LJ, UK; Division of Infection & Immunity, UCL Centre for Clinical Microbiology, University College London, Rowland Hill Street, London NW3 2PF, UK; Department of Infectious Diseases, Mycology Reference Centre Manchester, ECMM Centre of Excellence, Manchester Academic Health Science Centre, Wythenshawe Hospital, Manchester University NHS Foundation Trust, Manchester, UK; Division of Evolution, Infection and Genomics, Faculty of Biology, Medicine and Health, University of Manchester, Manchester, UK

## Abstract

The British Society of Antimicrobial Chemotherapy (BSAC) Academic Publishing Workshop, held at the Birmingham City Event Centre in the UK from 19 to 20 March 2025, provided practical guidance for early-career researchers and healthcare professionals seeking to understand and actively engage with the academic publishing process. This perspective extends the workshop’s expert insights to those who were unable to attend, summarizing key lessons across five thematic sessions.

The first session addressed journal selection, guiding participants on evaluating scope, impact metrics, open-access models and identifying predatory journals. The second explored peer-review models including single-blind, double-blind and open review, and reinforced reviewers’ role as guardians of research integrity. The third session focused on writing effective structured abstracts and developing resilience in managing desk and post-review rejections. The fourth provided a practical framework for converting conference poster presentations into full manuscripts. The fifth examined the evolving role of artificial intelligence in academic publishing, including its benefits for writing support and literature discovery, alongside ethical considerations. Complementing these sessions, hands-on peer-review exercises enabled participants to develop and apply concrete manuscript evaluation skills in a collaborative setting.

This perspective underscores that successful academic publishing demands continuous professional development, and that structured, expert-led training is essential for advancing research careers and maximizing scholarly impact.

## Introduction

Academic publishing presents significant challenges for researchers, especially early-career researchers (ECRs) and healthcare professionals in resource-limited settings. These researchers face disproportionate barriers to publishing in high-impact journals, must navigate complex submission processes, endure lengthy peer-review cycles and manage frequent manuscript rejections. Compounding these challenges, many ECRs often enter the publishing landscape with limited exposure on how to navigate the publishing process independently, having depended on Principal Investigators to manage much of the peer review and submission process during their training.^1^ These challenges often discourage authors from pursuing publication in reputable journals, thereby limiting the dissemination of valuable scientific knowledge.^2^

In response to these challenges, the British Society of Antimicrobial Chemotherapy (BSAC) convened an Academic Publishing Workshop (https://bsac.org.uk/academic-publishing-workshop/) from 19 to 20 March 2025, at the Birmingham City Event Centre in the UK. The workshop brought together participants from diverse professional backgrounds including doctors, early-career scientists, industry professionals, pharmacists and postgraduate students from across Africa, Australia, Europe, North America and the UK. This multidisciplinary and international representation enriched the discussions and ensured that the insights shared were broadly relevant across disciplines, career stages and geographic context.

**Figure 1.**
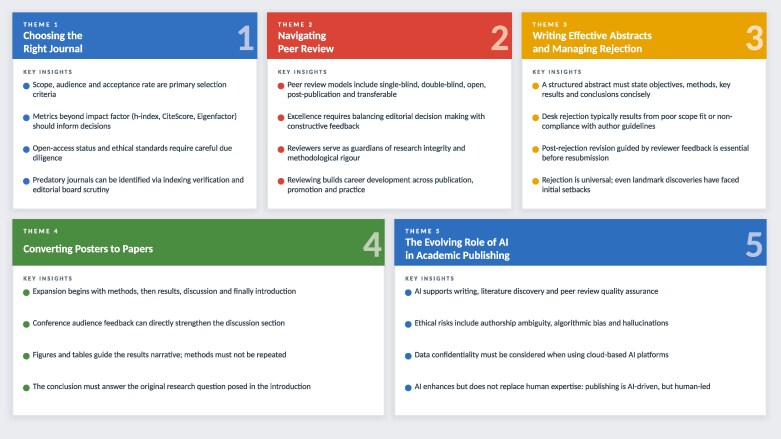
Five core themes for advancing scholarly publication skills among early-career researchers and healthcare professionals. The five core themes of the BSAC Academic Publishing Workshop 2025: key insights for early-career researchers and healthcare professionals. The British Society of Antimicrobial Chemotherapy (BSAC) Academic Publishing Workshop, held in Birmingham, UK (19–20 March 2025), addressed five core themes spanning the full scholarly publication cycle. These themes provide a comprehensive framework for building future academic publishing competency.

Here, we summarize insights from the workshop’s core themes: choosing the right journal, navigating peer review, writing effective abstracts, converting posters to papers and the evolving role of AI in academic publishing (Figure 1). Participant feedback collected following the workshop reflected positively on the sessions, with attendees particularly highlighting the practical guidance on journal selection, the peer-review exercises and the discussion on the ethical use of AI as the most impactful elements of the programme.

## Choosing the right journal for your work

A major challenge in academic publishing is choosing the right journal. This step is very crucial as it affects the visibility and credibility of one’s work and, if not done well, may prove to be a daunting and time-wasting task. Several key factors must be considered when selecting an appropriate journal. First, the scope and focus of the journal, which include its relevance, the type of articles accepted, the target audience and the journal’s acceptance rate, should be considered. A fast, yet thorough, peer review process that provides constructive feedback is also an important factor to consider. Researchers should note that the expected average time for peer review is often reported on the journal’s website and can serve as a useful benchmark when considering journals.

An essential part of due diligence involves looking at how material that has already been published in the target journal is performing. Key metrics to look for are citations of published works by other authors in high-impact factor journals and the usage or number of times the published works were downloaded. The altmetrics or alternative metrics of a journal should also be considered. Altmetrics are non-traditional measures of scholarly impact that track attention and engagement across different online sources, including social media shares, blog posts, news stories, HTML views, bookmarks and article downloads, tracked in real time by platforms such as Altmetric.com. Unlike citation metrics, which can take a longer time to accumulate, altmetrics offer quick insights into how publications are being received and engaged with by both the community of practice and non-academic audiences in real time.^3,4^ When selecting a journal, authors should consider the publication model, whether open access or subscription-based as this affects article visibility, article processing charges and funder compliance requirements. Publishing in open-access journals is particularly advantageous for researchers in resource-limited settings, as it increases the visibility and global reach of their work.^5^

Additional considerations include the ethical requirements of the journal, such as data integrity, conflict of interest and consent of any study participants. Authors should be aware of predatory and semi-predatory journals that send unsolicited and aggressive invitations to publish.^6^ A way to avoid falling foul to such predatory journals is to examine the journal’s editorial board and confirm it comprises reputable and credible experts in the field. A key takeaway from this session was the realization that authors should not excessively rely on the journal’s impact factor as the primary measure of research quality. Instead, they should also consider complementary indicators such as: the h-index, which reflects an individual author’s combined publication output and citation impact accessible via Web of Science or Scopus; the Citescore, which measures the average citations received per document published in a journal over a 4-year period, and the Eigenfactor score available at eigenfactor.org, which weights citations by the prestige of the citing journal.^7^ Each metric has recognized strengths and limitations as outlined in the San Francisco Declaration on Research Assessment,^8^ and researchers are encouraged to consult bibliometric guidance when interpreting them.

### Pitfalls and considerations in not-for-profit journals versus others

ECRs must be cautious when selecting a journal to publish their research work, as there are important ethical considerations associated with the publishing process. A not-for-profit journal is a society-owned journal published by academic or professional societies. They are driven by a mission to advance and support evidence-based literature rather than commercial gain. They are a reasonable and trustworthy option for ECRs. By contrast, for-profit journals are owned by publicly traded companies, private entities or individuals, and may prioritize revenue over research integrity. Some funder-owned journals may also self-publish or offer pre-print publication.^9^ Given these distinctions, ECRs are encouraged to prioritize not-for-profit journals where possible, as they are more likely to uphold rigorous editorial standards and align with the principles of responsible research dissemination.

A journal’s lack of adherence to ethical standards of publication can be a major pitfall for authors. Reputable journals require authors to confirm compliance with established authorship criteria, especially those of the International Committee of Medical Journal Editors, at the point of submission. The absence of requirements for transparent authorship declarations should be regarded as a warning sign.^10^ All authors must disclose any conflicts of interest, and data should be presented without fabrication or falsification. There should be no misrepresentation or exaggeration of findings and studies that do not support the desired conclusion should not be omitted. As part of research governance, authors must ensure their data is managed responsibly, including appropriate registration with ethics committees, storage and security. Authorship attribution equally requires careful consideration. Co-authorship should only be granted to individuals who have made substantial contributions to both the research process and manuscript writing, in line with International Committee of Medical Journal Editors criteria. Financial support, equipment provision or other industry contributions should be declared in the acknowledgements section rather than conferring authorship, unless the contributor’s involvement genuinely meets these criteria.

To avoid pitfalls associated with predatory journals, authors should look out for warning signs such as a lack of a transparent peer-review process, fake editorial boards, aggressive approaches to authors, unrealistic rapid publication timelines with promises of minimal revision, absence of indexing in major databases such as PubMed Central or Web of Science, vague or exorbitant article processing charges, and a general lack of impact credibility or impact factor. Journals can be verified through indexing in databases such as Web of Science and Beall's list.^11,12^ Think Check Submit, and the EQUATOR Network are additional resources that authors can employ in their arsenal against predatory journals. These web-based platforms assist authors in identifying trusted publishers for their research and provide guidance on writing and publishing high-impact health research.

## Excellence in peer review

Navigating the peer-review landscape requires more than technical knowledge. It demands ethical awareness and professional integrity. Understanding that peer review is the evaluation of scientific, academic or professional work by others working in the same field provides the foundation, but excellence lies in how this evaluation is conducted. The peer-review process follows five fundamental steps: research work on a specific subject, manuscript preparation, manuscript submission, journal peer review and feedback and, finally, if an article meets the editorial and peer standards, it is accepted and published.^13^ For ECRs, the strategic value of peer review extends beyond individual manuscripts through the four Ps framework: publication, presentation, promotion, and practice. Frequent reviewing builds publication experience and enhances writing skills. The skills developed transfer directly to evaluating conference presentations, academic promotion packets and practice applications.^14^ In addition, some journals, particularly those operating open peer-review models, often share a consolidated summary of all reviewer’s comments with the full reviewer group following an editorial decision, providing ECRs with a valuable opportunity to benchmark their our critique against that of more experienced peers.

Different peer-review models serve distinct purposes, and understanding these options helps reviewers choose appropriate approaches. Single-blind review in which the reviewer(s) identity is hidden remains the most common, while double-blind review in which the identity of both the authors and reviewers are hidden reduces bias by hiding both identities. Open peer review promotes transparency through public identities and comments, although this may inhibit objective feedback. Emerging models such as post-publication review and transferable peer review offer new engagement opportunities that forward-thinking researchers should consider.

Early-career authors may also be invited to contribute as peer reviewers, which broadens the pool of expertise available to the journal. Achieving excellence in peer review hinges on a fundamental pre-review checklist. Reviewers must possess sufficient subject expertise, declare any conflicts of interest, maintain complete impartiality and commit to providing timely feedback.

The best reviewers balance two critical objectives; contributing to editorial decision making through unbiassed evaluation, while improving manuscript quality through constructive feedback. They also serve as guardians of research integrity by alerting editors to potential plagiarism or duplicate submissions. Understanding and actively engaging in peer review represents both a professional obligation and a strategic career investment, essential for advancing science while developing transferable academic and professional skills. Overall, this session enhanced awareness of the principles of excellence in peer review and provided important concepts for those at the beginning of their journey in publishing and peer reviewing.

## Writing a strong abstract

An abstract serves as a succinct summary of an entire research paper, typically condensed into a single paragraph of around 250 words depending on the journal.^15^ It should be written in clear, accessible language while remaining comprehensive enough to encapsulate the key elements of the study. The study objectives should be clearly stated and aligned with the introduction. This ensures consistency in focus and scope of the abstract. A brief overview of the methods used, including the study population and general analytical approaches used should also be included. Only the results that directly address the stated objectives, and are supported by relevant data, should be included. Finally, a conclusion should logically follow on from the results and reinforce the study’s significance and any future work.

A well-structured abstract not only provides a snapshot of the research but also creates a compelling first impression. It positions the author to engage the reader and encourage further exploration of the full article. However, crafting a strong abstract requires practice and a good understanding of both the research content and the expectations of academic writing.

### Managing rejections

Rejection is a part of the publishing journey that every author will face at some point in their career. There are two types of rejection that can be issued; a desk rejection, where the editor declines the submission without peer review due to poor fit with the journal or a rejection after peer review. The latter is often issued when reviews are not sufficiently favourable for the editor to proceed with publication of the manuscript. Some of the reasons for manuscript rejection include unsuitability with journal’s scope, lack of novelty in the submitted works, inadequate data, plagiarism or non-compliance with ethical guidelines. Manuscript rejection can feel like a breaking point, however, rejection is normal and must be treated as an opportunity for growth. An encouraging example given during this session was that of Hans Krebs’s paper on the citric acid cycle, which was rejected by *Nature* in 1937 but later won the Nobel Prize. This shows that even groundbreaking work can face initial setbacks.^16^

To avoid rejection, researchers should carefully choose target journals and ensure their manuscript fits the scope of the journal. Carefully reading and following the target journal’s guidelines on submission will help avoid rejection at this stage. The manuscript should be formatted and proofread before submitting and authors should engage with the peer-review process once their work has been submitted. Researchers should also consult the journal’s aims and scope to identify and submit under the appropriate article category as submitting under an incorrect article type is a frequent and avoidable cause of desk rejection. Strict adherence to the journal’s recommended word limits, structure, accurate citations and reference formatting is equally essential. A compelling cover letter outlining key findings may improve chances of acceptance for peer review.

When it comes to the manuscript itself, writing clearly while maintaining high language standards is essential. Submitted works should be substantial and advance the field, rather than composed of fragmented research pieces. Conclusions should be supported by the findings presented, avoiding speculation or exaggeration of data with study limitations noted accurately. Each paragraph and/or section must be, as much as possible, ‘the natural follow-up’ to the previous one to maintain a smooth flow and keep the reviewer (and later the reader) interested and focused. If the same manuscript is rejected multiple times by different journals, it is important to take time to review it in detail and reflect on the feedback provided. It may be necessary to make fundamental changes to the work before attempting to submit it again.

## From poster to paper

Poster presentations play a pivotal role in ECRs’ development by providing networking opportunities, early feedback and increased visibility at conferences. The real challenge lies in converting visual summaries into a thorough but focused literature review, comprehensive methodology, detailed analysis of results and meaningful conclusions. Although daunting, a poster can be converted to a well-written, well-cited and journal-compliant research paper by following a systematic approach.

Transforming a poster into a manuscript begins with reminding yourself about the aim of the study and then expanding the materials and methods section into a comprehensive study design paragraph that outlines the procedures, sample sizes, tests performed and timelines of work completed with clearly defined terminology and grouping criteria. This is followed by sections on results, discussion, introduction and conclusion in that order. This sequence is intentional. Beginning with methods and results assures that same terminology and abbreviations are used in figures, tables and text, and anchors the writing process in the empirical evidence, ensuring the discussion is shaped by what the data show. The results section should exploit poster feedback to identify and clarify confusing elements, allowing figures and tables to guide the narrative while presenting only analysed data showing statistical significance where relevant. Repeating methods or interpreting findings must be avoided.

The discussion should open with a summary of the main outcomes of the study, bringing various results together without repeating the results summary. Findings should then be presented within the broader research context and compared with similar published studies. However, the discussion should not repeat content from the introduction or serve as a secondary literature review. Authors should also consider whether results can be extrapolated to different settings. A balanced assessment of the limitations of the study and methods used should follow, alongside suggestions for future research where appropriate. Finally, the conclusion should be a snapshot of the overall findings and key takeaways that address the fundamental questions highlighted in the introduction.

Writing the introduction last guarantees it is precisely framed around the research question the completed manuscript ultimately answers, and what the reader needs to know to understand what was done and why. In the final manuscript, the introduction precedes the material and methods section, to address four fundamental questions: What is the problem? What is already known? What gaps remain? And, therefore, what does this study aim to investigate? It should clearly state the research objectives and incorporate recent studies. Finally, check the abstract you used for the poster, and make sure the terminology, abbreviations, details of the results and conclusions match with the text.

## The future of AI in academic publishing

Since November 2022, when OpenAI’s ChatGPT brought large language models into mainstream use, academics have been grappling with the question of the ethical application of AI in academic research. With >3 million papers published annually worldwide, AI has emerged as a game changer in academic publishing, transforming research writing, analysis, review and dissemination.^17^

AI applications in academic publishing span three broad areas. These include tools that support manuscript preparation and language editing, systems that facilitate literature searching and citation management through natural language processing, and publisher-deployed platforms that assist with plagiarism screening, data integrity checking and peer reviewer identification.^6,18,19^

However, the integration of AI raises several ethical concerns that warrant careful consideration. AI authorship raises questions about intellectual ownership. Bias risks emerge when AI systems perpetuate existing inequalities in research representation. Plagiarism concerns are intensifying as the distinction between AI-supported writing and independent scholarly thought becomes less defined. Two further risks deserve particular attention: first, data confidentiality, given that submitting unpublished manuscript content to commercial AI platforms may compromise sensitive research data; and second, research integrity, as AI systems can generate inaccurate or entirely fabricated references, a phenomenon termed ‘hallucination’, which, if undetected, poses a direct threat to the reliability of the published literature.^6,20^

Despite these challenges, these considerations are essential for ensuring fair access to knowledge and maintaining academic integrity. The goal is to enhance scholarly communication and research quality, not to replace the critical thinking and creativity that researchers bring to their work. AI should be understood as a collaborative instrument that amplifies, rather than replaces, human expertise. The future of academic publishing is AI-driven, but human-led.^18^

The 2-day workshop culminated with a hands-on peer-review exercise during which participants analysed manuscripts published in the *Journal of Antimicrobial Chemotherapy* (*JAC*). This exercise provided first-hand experience of reviewing scholarly articles, enabling participants to identify the key aspects needed to enhance the impact of any published work.

### Conclusion

The BSAC Academic Publishing Workshop offered practical, career-relevant lessons across five interconnected themes: selecting the right journal, excellence in peer review, crafting effective abstracts, converting posters to full manuscripts and adopting AI tools responsibly. Collectively, these insights highlight that successful academic publishing requires deliberate, continuous professional development, encompassing a commitment to learning new skills, embracing evolving best practices and upholding responsible research conduct. ECRs and healthcare professionals are encouraged to translate these insights into immediate action. Practical next steps include actively seeking peer-review opportunities with journals, using Think Check Submit to evaluate journals before submission, consulting the EQUATOR Network for reporting guidelines relevant to their study design, practising structured abstract writing using author guidelines of their target journal and actively engaging with AI tools responsibly. Participating in future BSAC workshops and similar capacity-building events remain strongly encouraged as investing in one’s publishing literacy is fundamental to advancing both individual research careers and sustaining the integrity of global scientific discourse. Future iterations of this workshop would benefit from expanding the curriculum to include topics such as patient and public involvement in research, which is increasingly recognized as essential for ensuring that research is relevant, inclusive and aligned with community needs.
